# Long Non-coding RNA DANCR as an Emerging Therapeutic Target in Human Cancers

**DOI:** 10.3389/fonc.2019.01225

**Published:** 2019-11-15

**Authors:** Shi-Jia Jin, Ming-Zhu Jin, Bai-Rong Xia, Wei-Lin Jin

**Affiliations:** ^1^Key Laboratory for Thin Film and Microfabrication Technology of Ministry of Education, Department of Instrument Science and Engineering, Shanghai Engineering Center for Intelligent Diagnosis and Treatment Instrument, School of Electronic Information and Electronic Engineering, Institute of Nano Biomedicine and Engineering, Shanghai Jiao Tong University, Shanghai, China; ^2^Shanghai Jiao Tong University School of Medicine, Shanghai, China; ^3^Department of Gynecology, The Affiliated Tumor Hospital, Harbin Medical University, Harbin, China; ^4^National Center for Translational Medicine, Collaborative Innovational Center for System Biology, Shanghai Jiao Tong University, Shanghai, China

**Keywords:** long non-coding RNA, cancer, DANCR, mechanism, therapy

## Abstract

Long noncoding RNAs (lncRNAs) are emerging as important regulators of numerous biological processes, especially in cancer development. Aberrantly expressed and specifically located in tumor cells, they exert distinct functions in different cancers via regulating multiple downstream targets such as chromatins, RNAs, and proteins. Differentiation antagonizing non-protein coding RNA (DANCR) is a cytoplasmic lncRNA that generally works as a tumor promoter. Mechanically, DANCR promotes the functions of vital components in the oncogene network by sponging their corresponding microRNAs or by interacting with various regulating proteins. DANCR's distinct expression in tumor cells and collective involvement in pro-tumor pathways make it a promising therapeutic target for broad cancer treatment. Herein, we summarize the functions and molecular mechanism of DANCR in human cancers. Furthermore, we introduce the use of CRISPR/Cas9, antisense oligonucleotides and small interfering RNAs as well as viral, lipid, or exosomal vectors for onco-lncRNA targeted treatment. Conclusively, DANCR is a considerable promoter of cancers with a bright prospect in targeted therapy.

## Introduction

**Figure 1:**
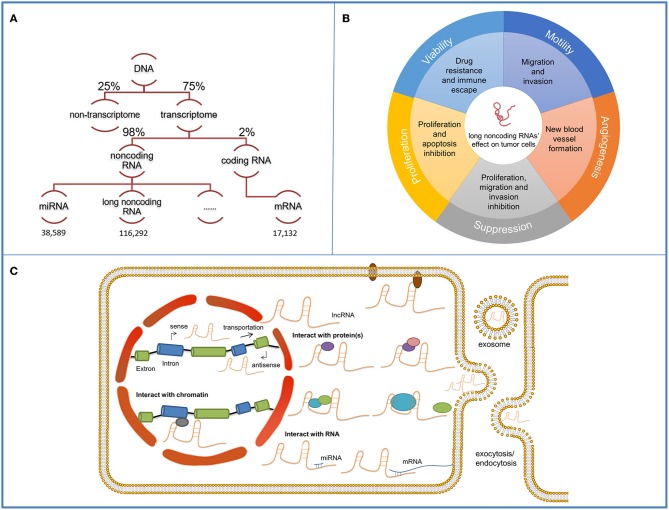
Functions and mechanisms of long non-coding RNAs (lncRNAs). The schematic illustrates lncRNAs and their functions and mechanisms in tumor cells. **(A)** Among the 75% actively transcribed sequences in the human genome, 98% are non-coding sequences where long non-coding sequences occupy the majority. The quantity of miRNAs, lncRNAs and miRNAs mapped on the schematic are obtained from the respective databases. **(B)** LncRNAs' functions on cancer development contain viability, motility, angiogenesis, proliferation promotion, and suppression. **(C)** LncRNAs have distinct working compartments. They can assemble with nucleus proteins and regulate transcription by interacting with the histone proteins or the promoter regions. They can also transmit to the cytoplasm and participate in signaling transmission by mediating the modification of components in cellular pathways. They can be contained in exosomes and delivered for intercellular communication. Mechanically, they can interact with protein/protein complex and form protein-RNA complex, serve as a scaffold and promote the assembling of proteins or complementary bind to RNAs to exert their functions.

Approximately 75% of the human genome can be transcribed into RNAs, yet no more than 2% encodes proteins (1). Non-coding RNAs occupy a large part of the human transcriptome and long non-coding RNAs (lncRNAs) [transcripts over 200 nt in length and lack the potential of coding protein (2)] are the major components (Figure 1A) (3). Extensive research on lncRNAs has been conducted for several decades and considerable progress has been achieved in this field. H19 is the first recognized lncRNA that can be transcribed from both protein coding and non-coding DNA (4). Its deficit in open reading frame and inactive interaction with ribosomes distinguish it from normal protein-coding mRNAs and extend human understanding of RNA transcriptome (5). X-inactive specific transcript (Xist) is one of the most well-known lncRNAs which is associated with the silencing of X chromosome (6). With the technical improvement of gene sequencing technology (7), the past 30 years have led to the discovery of a wide spectrum of evolutionally conserved and tissue-specific lncRNAs. They are located in cell nucleus and cytoplasm and play an important role in pathophysiological processes (8). Their various functions (promote tumor proliferation, motility, viability, and angiogenesis) in cancer regulation becomes a research focus in recent years (9) (Figure 1B). Mechanically, by interacting with proteins and RNAs, lncRNAs can regulate the transcription and post-transcription of RNAs or mediate the modification of proteins to adjust the cellular signaling pathways. LncRNAs have also been involved in the clinical cancer treatment. For example, prostate cancer antigen 3 has been approved by the Food and Drug Administration (FDA) as a biomarker in urine for prostate cancer diagnosis; it displays a better diagnostic effect compared with traditional serum prostate-specific antigen testing (10). Some lncRNAs, such as lncRNA Hox Transcript Antisense Intergenic RNA (HOTAIR) in breast cancer, are proven to enhance cells' resistance to certain drugs, which can guide drug selection in cancer chemotherapy (11). Onco-lncRNA targeted therapy is a novel and promising therapeutic method with collective efficacy, high specificity and few side effects (12). Many synthetic therapeutic products have been constructed and pre-clinical experiments have been conducted to promote the clinical application of lncRNA-targeted therapy.

Due to the overwhelming quantity of lncRNAs in the human genome and the lack of methods for predicting their functions, massive work is still needed to better understand the lncRNAs. LncRNA differentiation antagonizing non-protein coding RNA (DANCR) can serve as a paradigm for lncRNA investigation. DANCR is a typical oncogenic lncRNA which exerts multiple functions in cancer development. It has also demonstrated its value in cancer therapy as a promising target (12).

This article summarized the functional properties and mechanisms of DANCR in tumor progression. The general targeted therapies against onco-lncRNAs were introduced and the specific methods to target DANCR were discussed. This article can provide referential value for latter onco-lncRNA investigations and insights into the clinical treatment against cancers caused by aberrant expression and regulation of lncRNAs.

## DANCR: A Critical onco-lncRNA

DANCR also named as Anti-Differentiation Non-coding RNA (ANCR), Small nucleolar RNA host gene protein 13(SNHG13), upregulating transcripts 2 (AGU2) is an oncogenic lncRNA located on chromosome 4 (13, 14). It was first identified as a transcript of unknown function whose expression is dramatically upregulated during adipogenesis of pluripotent human mesenchymal stem cells (hMSCs) (13). Later it was recognized as an anti-differentiation non-coding RNA as it promoted epidermal progenitor cell maintenance and prevented differentiation in osteoblasts (15, 16). Extensive data have shown that DANCR correlates with the prognosis of various human cancers, and exerts diverse oncogenic functions via multiple downstream targets. It will serve as a novel target for targeted therapy.

### DANCR: A Biomarker of Tumor Progression and Prognosis

The discovery that DANCR over-expressed in colorectal cancer promoted tumor progression and predicted poor prognosis introduced the study of DANCR into the field of cancer (17). DANCR can serve as a novel biomarker of CRC progression, and its high expression is correlated with TNM stage, histologic grade, and lymph node metastasis (17). In addition, a recent meta-analysis and systemic review illustrated that DANCR expression is of significant prognostic value in various cancers (18). It is discovered that high lncRNA DANCR expression is associated with shorter overall survival and disease-free survival, predicting worse prognosis (18).

In the wake of the deepening of the research, DANCR was found to be aberrantly expressed in numerous cancers and exerted multiple regulating functions. The underlying mechanisms were also being constantly elucidated.

### Multiple Functions of DANCR in Cancers

DANCR is a typical oncogenic lncRNA, overexpressed in various tumor cells. It can regulate the progression of numerous cancers by modulating the expression or activity of downstream targets and consequently promoting hallmarks of cancer including cell proliferation, cell motility, tumor angiogenesis, and cell viability.

#### Cell Proliferation

In hepatocellular carcinoma, it is observed that down-regulated DANCR expression can boost cell apoptosis and cell cycle block in G1 which underlies that DANCR can promote tumor cell proliferation by inhibiting cell apoptosis and facilitating growth inhibition evasion (19). DANCR overexpressed in endometrial carcinoma can alleviate the inhibitory effect of miR-214 on target mRNAs and inhibit cell apoptosis and promote proliferation (20). In MYC-induced lymphoma, DANCR limits the transcription of Cell-cycle inhibitor p21 (CDKN1A) which can cause cell cycle arrest by restricting the activity of cyclin-dependent kinases. Inhibition of CDKN1A leads to accelerated G1/S shift and promote the proliferation of lymphoma cells (21, 22).

#### Cell Motility

Cell motility refers to the metastasis and invasion properties of cells and malignant cells to vital organs and it is the major cause of cancer deaths (23). DANCR can promote tumor cell motility in various ways.

DANCR promotes the motility of nasopharyngeal carcinoma cells by stabilizing hypoxia inducible factor-1α, the subunit of Hypoxia inducible factor 1 (HIF-1) which are transcription factors that can activate several hypoxia-responsive genes in response to hypoxia microenvironment and promote the metastasis and invasion of cancer cells (24, 25). Matrix Metallopeptidase 16(MMP16) is one of the extracellular matrix (ECM) enzymes which can decompose the ECM physical barriers and promote the metastasis of cells (26). MMP16 can activate MMP2 for collagen degradation (27). DANCR can upregulate MMP16 protein and lead to enhanced motility of pancreatic cancer cells (28). Another promoting factor of carcinoma metastasis is Epithelial-mesenchymal transition (EMT), during which epithelial cells lose their epithelial characteristics and cell-cell contact, thus increasing their invasive potential and the migratory capacity and invasive potential of tumor cells (29). DANCR can modulate the EMT progression by upregulating Rho-associated coiled-coil containing protein kinase 1 (ROCK1) and promote metastasis in cervical cancer (30).

#### Tumor Angiogenesis

Tumor angiogenesis is one of the hallmarks of cancer which provide essential nutrients and oxygen to proliferating cancer cells (31). DANCR can increase vascular endothelial growth factor A (VEGF-A) expression, mediate vascular permeability as well as the multiplication and motion of endothelial cells and promote the neovascularization in ovarian cancer cell (32, 33).

#### Cell Viability

The innate or acquired chemo-resistance of tumor cells is a main problem in the clinical cancer therapy. DANCR promotes the docetaxel resistance in prostate cancer cells by mediating the upregulation of multidrug resistance associated protein (MDR) and p-glycoprotein (P-gp), which bump many chemotherapeutic drugs out of the cells and limit the intracellular drug dosage to sub-therapeutic levels (34, 35). DANCR can also reduce the sensitivity of glioma cells to cisplatin through PI3K/Akt signaling pathway activation and mediate the recruitment of NF-κB protein to drug resistance genes for their enhanced transcription (36).

Generally, DANCR exerts multiple regulatory functions on tumor progression of numerous cancers, revealing its role as an important pro-tumor regulator (Table 1). And notably, DANCR, an oncogenic lncRNA in most cases, suppresses proliferation and motility and promotes apoptosis in renal carcinoma cells (RCCs), its mechanism remains unclear (61).

**Table 1:** Functions of DANCR in human cancers.

**Cancer type**	**DANCR expression**	**Functional properties (validated)**	**References**
Retinoblastoma	↑	Tumor proliferation, motility	(37)
Nasopharyngeal carcinoma	↑	Tumor proliferation, motility	(25, 38)
Esophageal squamous cell carcinoma	↑	Tumor proliferation, motility	(39)
Non-small-cell lung cancer	↑	Tumor proliferation, motility	(22, 40, 41)
Triple negative breast cancer	↑	Tumor proliferation, motility	(42, 43)
Hepatocellular carcinoma	↑	Tumor proliferation, motility	(44)
Cholangiocarcinoma	↑	Tumor proliferation, motility	(45)
Pancreatic ductal adenocarcinoma	↑	Tumor proliferation, motility	(46)
Pancreatic cancer	↑	Tumor proliferation, motility	(28, 47)
Cervical cancer	↑	Tumor proliferation, motility	(30, 48)
Ovarian cancer	↑	Tumor proliferation, motility, angiogenesis	(33, 49)
Colorectal cancer	↑	Tumor proliferation, motility	(50)
Bladder cancer	↑	Tumor proliferation, motility	(51)
Osteosarcoma.	↑	Tumor proliferation, motility	(52)
Gastric cancer cells	↑	Tumor proliferation, motility, viability	(53, 54)
Glioma	↑	Tumor proliferation, motility, viability	(36, 55, 56)
Endometrial carcinoma	↑	Tumor proliferation	(20)
Lymphocyte carcinoma	↑	Tumor proliferation	(57)
Prostate cancer	↑	Tumor motility, viability	(34, 58, 59)
Papillary thyroid cancer	↓	Tumor suppression	(60)
Renal cell carcinoma	↓	Tumor suppression	(61)

### Common Downstream Targets of DANCR

LncRNAs perform their multiple regulatory functions on tumors by interacting with numerous downstream targets (Figure 1C). The downstream targets include proteins and RNAs. LncRNAs can bind with histone-regulating proteins [such as Enhancer of zeste homolog 2 (EZH2), histone deacetylase (HDAC)], which catalyze the histone protein in chromatins, and the promoter-binding proteins, which can bind to the promoters or enhancers of certain genes, and guide the silencing of certain genes (34, 62, 63). They can interact with cytoplasm proteins, mRNAs and miRNAs and thus modulate the cells via stabilizing, regulating and modifying the components within signaling pathways (64). LncRNAs can also bind with integrated proteins and mediate the transmission of the signaling received from the extracellular environment (64). DANCR, as a typical lncRNA, exerts its regulatory functions mainly via protein-DANCR and miRNA-DANCR interaction (Table 2). Herein, we review some common targets of DANCR which are discovered to interact with DANCR in multiple cancers and further discuss the role of DANCR in particular cancers to better understand the regulatory mechanism of DANCR.

**Table 2:** The putative molecular mechanisms of DANCR.

**Cancer type**	**DANCR-binding miRNA**	**Downstream target of miRNA (validated)**	**References**
**DANCR INTERACTS WITH miRNAs**			
Glioma	miR-634	RAB1	(56)
	miR-216a	LGR5	(65)
Esophageal squamous cell carcinoma	miR-33a-5p	ZEB1	(39)
Non-small cell lung cancer	miR-758-3p	–	(22, 50)
	miR-216a	EIF4B, JAK2, MALAT1	
Breast cancer	miR-216a-5p	Nanog, OCT4 and SOX	(42)
Hepatocellular carcinoma	miR-216a-5p	KLF12	(19)
	miR-27a-3p	LIMK1	(66)
Pancreatic ductal adenocarcinoma	miR-33a-5p	AXL	(46)
	miR-214-5p	E2F2	(47)
Pancreatic cancer	miR-33b	MMP16	(28)
	miR-135a	NLRP3	(67)
Cervical cancer	miR-665	TGFBR1	(48)
	miR-335-5p	ROCK1	(30)
Endometrial carcinoma	miR-214	–	(20)
Ovarian cancer	miR-145	VEGF	(33)
Colorectal cancer	miR-577	HSP27	(68)
Bladder cancer	miR-149	MSI2	(69)
Prostate cancer	miR-135a	–	(59)
	miR-34a-5p	JAG1	(34)
	miR-214-5p	E2F2	(47)
Osteosarcoma	miR-33a-5p	AXL	(54)
	miR-1972p		
**Cancer type**	**DANCR-interacting mRNA**	**Competitive miRNA**	**References**
**DANCR INTERACTS WITH mRNAs**			
Retinoblastoma	MMP-9	miR-34c and miR-613	(37)
Hepatocellular carcinoma	CTNNB1	miR-214, miR-320a, miR-199a	(70)
Cervical cancer	TGFBR1	miR-665	(30)
**Cancer type**	**DANCR-interacting protein**	**Consequence**	**References**
**DANCR INTERACTS WITH proteins**			
Nasopharyngeal Carcinoma	NF90/NF45	Stabilizing HIF-1-α	(25)
	STAT3	Enhancing IL-6/JAK1/STAT3 signaling.	(38)
Lymphatic carcinoma	CDKN1A	Limited expression of p21	(57)
Non-small lung cancer	EZH2	Silencing promoter of p21	(22)
Triple negative breast cancer	RXRA	Activating serine phosphorylation of RXRA and upregulates PI3K/AKT	(71)
	EZH2	Promote transcription of CD44 and ABCG2	(43)
Cholangiocarcinoma	EZH2	Silencing promoter of FBP1	(45)
Gastric cancer	EZH2, HDAC3	Silencing lncRNA-LET	(72)
Prostate cancer	EZH2	Silencing TIMP2/3-promoter	(58)

EZH2 is a common target protein of DANCR. Mechanically, EZH2 enables the silencing of gene expression by catalyzing histone H3 trimethylation at lysine 27 (H3K27me3) in target gene promoters (73). DANCR can bind with EZH2, guide it to silence the specific gene and thus regulate the cells. EZH2-DANCR interaction was first discovered in human fetal osteoblastic cell where the lncRNA-protein complex inhibited Runt-related transcription factor-2 expression and pursuant osteoblast differentiation (16). EZH2-DANCR mechanism can also be found in many cancers: In non-small-cell lung cancer cells, it promotes cell proliferation, migration, and invasion by inhibiting p21 expression (22). In gastric cancer cells it enhances cell migration and invasion through suppression of lncRNA-LET (Low Expression in Tumor) (72). In prostate cancer cells it boosts cell invasion by epigenetically silencing expression of tissue inhibitors of metalloproteinase 2/3 (58). In cholangiocarcinoma it regulates proliferation and migration by epigenetically silencing fructose-1,6-bisphosphatase 1. By silencing the tumor inhibitory gene via EZH2 recruitment, DANCR manages to promote cancer progression (45).

MicroRNAs are small regulating RNAs (−22 nt in length) derived from the short hairpin regions of RNA transcripts and they induce cleavage and suppression of translation via complementary binding to target RNAs (74). DANCR can acts as competitive endogenous RNA (ceRNA) and competitively bind tumor suppressive miRNAs to restore the functions of target oncogenic mRNAs. miR-216a is a common tumor suppressive miRNA and downstream target of DANCR. In lung cancer, DANCR induces cell proliferation and colony formation via sequestering miR-216a-5p (75). In breast cancer, DANCR targets miR-216a-5p, increases Nanog, SOX2, and OCT4 and promotes cell proliferation and invasion (42). In glioma, DANCR can restore LGR5 (Leucine-rich repeat-containing G protein-coupled receptor 5), PI3K, AKT, and p-AKT accumulation reduced by miR-216a, facilitating proliferation, migration, invasion, and angiogenesis and inhibited apoptosis (65). The massive targets of miR-216a complicate the functions of DANCR in cancer cells. In some cases, a novel DANCR-mRNA-miRNA interacting pathway is observed. For instance, DANCR competitively sponges the 3′UTR of transforming growth factor beta receptor 1 (TGFBR1) mRNA in cervical cancer cells, which blocks miR-665 from binding to and degrading TGFBR1 and promotes the TGFBR1-mediated oncogenic ERK (extracellular regulated protein kinases)/SMAD pathway (48).

### Detailed Roles and Molecular Mechanisms of DANCR in Different Cancers

**Figure 2:**
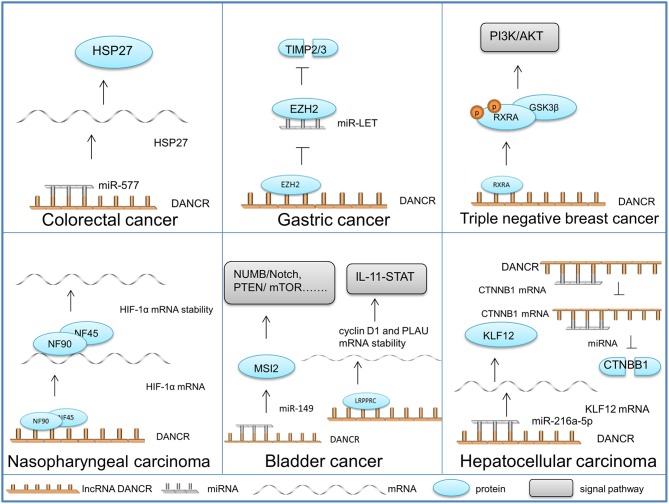
Functional properties and molecular mechanisms of DANCR in different cancers. The schematic illustrates the mechanisms of DANCR in different cancers.

In this section, the role of DANCR in particular cancers is discussed so as to clarify the molecular mechanisms of DANCR in a more specific way and instruct the therapy of DANCR aberrantly-expressed cancers (Figure 2).

#### Colorectal Cancer

DANCR acts as a miRNA sponge in colorectal cancer (CRC) cells, which is the most common role that DANCR plays in cancer development. Harboring the same miR-577 binding site, DANCR and heat shock protein 27 (HSP27) mRNA competitively bind to miR-577. The overexpression of DANCR in CRC cells alleviates the degradation impact of miR-577 on HSP27, which contributes to cell proliferation and metastasis (50).

#### Gastric Cancer

DANCR activated by SALL-4 (Sal-like protein 4) (53) mediates the EZH2-HDAC3 (histone deacetylase 3) complex to regulate the transcription of lncRNA-LET. It binds to the protein complex and directs it to the promoter of LET (Low Expression in Tumor), thereby silencing the tumor suppressive RNA and promoting tumor metastasis (72).

#### Nasopharyngeal Carcinoma

HIF-1α, generally known as the core regulator of the hypoxic response, has been validated as a critical positive regulator of nasopharyngeal carcinoma (NPC) cell metastasis. DANCR can combine the NF90 (nuclear factor 90)/NF45 (nuclear factor 45) complex with its AU-rich elements (AREs) in 3′ region, which stabilizes HIF-1α mRNA and upgrades the motility of NPC cells (25).

#### Triple Negative Breast Cancer

In triple-negative breast cancer (TNBC), DANCR functions by mediating protein assembly and modification. DANCR can bind with the phosphorylation site of RXRA, recurring glycogen synthase kinase 3 beta (GSK3β) to sponge RXRA and promote RXRA ser phosphorylation. The structural change in RXRA suppresses its interaction with the promoter of phosphatidylinositol-4,5-biophosphate 3-kinase catalytic subunit alpha (PIK3CA), thereby enhancing PIK3CA in both mRNA and protein levels. Consequently, protein PIK3CA activates the P13K/AKT pathway, leading to the proliferation and tumor growth of TNBC (71).

#### Bladder Cancer

Bladder cancer (BC) whose proper diagnosis and efficient treatment are current challenges in urology, is the most frequent malignancy of the urinary tract (76). The following two mechanisms of DANCR are discovered in BC cells: DANCR can combine with the miRNA-149 in BC cell cytoplasm, positively regulating the expression of Musashi RNA binding protein 2 (MSI2). Elevated MSI2 protein enhances the transcription and translation of the essential components in the oncogenic signaling pathways, such as NUMB/Notch, PTEN (Phosphatase and tensin homolog)/mTOR, TGF-β (transforming growth factor-β)/SMAD3, MYC, and cMET (77). DANCR can also direct a leucine-rich pentatricopeptide repeat containing (LRPPRC) to stabilize mRNA, thereby increasing cyclin D1 and PLAU (Plasminogen Activator, Urokinase) expression levels and activating IL-11 (Interleukin 11)-STAT3 (Signal Transducer and Activator of Transcription 3) signaling. Through the mechanisms above, DANCR facilitates the proliferation and invasion of BC cells (51).

#### Hepatocellular Carcinoma

Similar to CRC, oncogenic DANCR within hepatocellular carcinoma (HC) has also been found to have multiple regulating targets. KLF12 (Krueppel-like factor 12) can be positively regulated by DANCR through miR-216a-5p sponging, leading to malignant progression (19). DANCR can also competitively bind to the 3′UTR of Catenin beta-1 (CTNNB1) to block miRNA-mediated CTNNB1 suppression, leading to the initiation and progression of HC (78).

We can conclude that DANCR can regulate the signaling pathways by alleviating miRNA's inhibitory functions on target RNA, combining with proteins and guiding them to modulate the expression or the stability of RNAs or facilitating the integration and subsequent modifying of proteins. Moreover, DANCR can manipulate more than one target in cancer and join in multiple pathways. As we can see, due to the diverse mechanisms of DANCR and limited research on it, it is hard to foresee the unknown mechanisms and functions of DANCR in cancers. However, lncRNA's collective involvement in the pathways of numerous cancers does make it a promising target for cancer therapy.

## Potential Therapeutic Approaches Targeting DANCR

**Figure 3:**
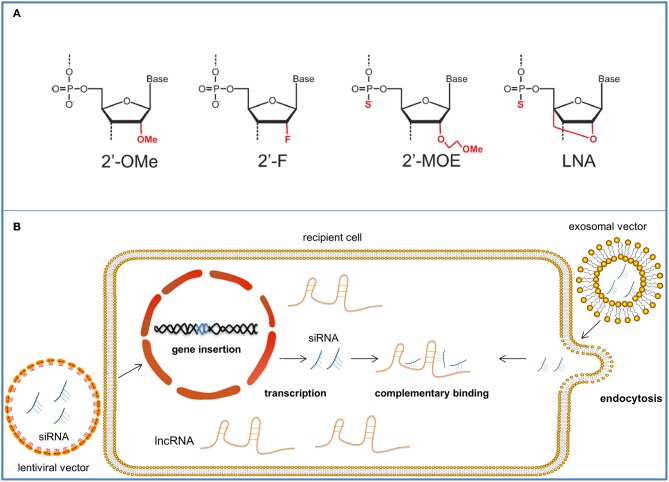
The therapeutic approaches targeting onco-lncRNA—DANCR. The schematic illustrates several targeting methods against onco-lncRNAs by using LNAs or siRNAs for lncRNA silencing and degradation. **(A)** Antisense oligonucleotides can be chemically modified to have strengthened affinity and stability. The figure shows four common modifications of ASOs. **(B)** SiRNAs are packaged in nanoparticle vectors for improved targeting. Lentiviral vectors can reverse and insert the siRNA sequence into the genome of target cells and achieve stable transfection. After transcription, siRNAs complementarily bind the target onco-lncRNAs and suppress their tumor promoter functions. Exosomal nanovectors can directly deliver the siRNAs to the target cell cytoplasm for onco-lncRNA inhibition through endocytosis.

The potency of lncRNA-based cancer therapy has been pointed out in previous years. Targeting lncRNAs for cancer treatment is a novel and promising therapy due to their collective therapeutic efficacy, high specificity, and few side effects (12). The possible molecular drugs against lncRNAs and nanoparticle vectors for their delivery are as follows (Figure 3).

### CRISPR/Cas9

The CRISPR/Cas9 technology can be used to target the endogenous loci of lncRNAs and restrict their transcription (79). The CRISPR system discovered in many bacteria as an immune mechanism for invading nucleic acid elimination can produce CRISPR RNAs (crRNAs) with certain “protospacer” regions complementary to the foreign DNA site (80). The crRNAs together with transactivating CRISPR RNAs (tracrRNAs) form the RNA pairs which combine and direct Cas9 nuclease to cleave complementary target-DNA sequences and cause DNA double-stranded break (DSB) (80). The CRISPR/cas9 system can be applied for target DNA cut and gene knockout via a designed single-guide RNA (sgRNA) complementary to the target gene (81). Progress has been made in the field: Cas9 nickases can cut one strand rather than both strands of the target DNA site (82). Catalytically inactive Cas9 (dCas9) enables the transcription regulation of target genes which may partly relieve the safety concern about irreversible frameshift disruptions caused by DNA cut (83). The dCas9 protein can be recruited to the coding region of genes and sterically block their transcription (84). Moreover, dCas9 can be fused to protein domains capable of recruiting repressive chromatin-modifying complexes, and lead to the silencing of particular genes determined by the engineered sgRNA (84). Recently, by targeting Erb-B2 Receptor Tyrosine Kinase 2(HER2) gene, CRISPR-Cas9 has managed to inhibit cell proliferation and tumorigenicity in HER2-positive breast cancer cells, demonstrating its potency in clinical application (85). The CRISPR/Cas9 technology has also been experimented for targeting oncogenic lncRNAs and further improvement is in demand for better efficacy (86).

### Antisense Oligonucleotides

Antisense oligonucleotides (ASOs) can be used in lncRNA-targeted therapy. They are synthetic and modified oligonucleotide single strands that can complementarily bind to certain sequences of their target RNAs. ASOs exert their inhibiting effect mainly by recruiting ribonuclease H (Rnase H) to degrade the targets. They comprise complementary DNA single strands to form DNA/RNA duplexes for Rnase H recognition and later cleavage (87). Vulnerable to various existing ribonucleases (88), ASOs are unstable *in vivo*. Combined with their relatively high charge and hydrophilicity (89), all these characteristics make crossing membranes of targeted cells difficult. Similar to other RNA therapeutic products, ASOs must be chemically modified to improve their pharmacological properties. Phosphorylation is the most commonly employed modification method (88) that involves substituting the phosphodiester backbones of the oligonucleotide strands for phosphonothioate (Ps) linkages (87). In this way, nucleotides become more hydrophobic and have higher adhesion to plasma proteins, enabling them to enter the targeted cells (68). Additionally, their resistance against nucleuses is advanced, making the products more stable (90). However, phosphorylation can reduce ASO's affinity to RNA and increase the likelihood of non-specific protein binding (90), which is harmful to regulation. In addition to phosphorylation, other widely used modifications include 2′ sugar modifications [such as 2′-O-methyl, 2′-fluoro (2′-F) and 2′- O-(2-methoxyethyl)(2′-MOE)],which all result in high affinity to RNA along with their diminished or disappeared ability to attract Rnase H (91). Uniformly modified ASOs are ASOs that completely lose their ability to recruit Rnase H for degradation, and they can sterically block certain domains to inhibit further interaction (92). Among all these synthetic products, locked nucleic acid (LNA), which is produced by changing the 20-hydroxy (20-OH) for 20,40-O-methylene bridge, exhibits the best affinity to RNAs (93). LNA can be used as the flank of GAPMER, which is a widely-used artificial complex for RNA targeting (88). The center of GAPMER is a DNA monomer which can enable Rnase H cleavage of the targeting mRNA, while LNA flanks increase its affinity to RNA and resistance to nuclease (69). Both miRNA affinity and nucleotide stability are achieved in this modification. Nusinersen, a splicing switching ASO, is a successful application of ASO in clinical treatment which was approved by the FDA in 2016 and became the first drug to treat spinal muscular atrophy (94). And to achieve better pharmaceutical effect, considerable work has to be done to achieve specific transportation (95).

### Small Interfering RNAs

We can also use small interfering RNAs (siRNAs) to target lncRNAs. In 2018 patisiran became the first approved RNAi drug to remedy hereditary transthyretin amyloidosis (70). SiRNAs are usually chemically synthesized or evolved from DNA-based small hairpin RNA (shRNA) (96). And compared to chemically synthesized siRNAs, effective shRNA expression DNA sequence could retain gene silencing for a longer period (97). Mechanically, siRNAs induce gene silencing by complementing with the target RNA and forming an RNA-induced silencing complex to splice and degrade the target (98). SiRNAs can be chemically modified to improve stability and prevent nuclease degradation. For example, boranophosphate siRNAs were 10 times more nuclease resistant than unmodified siRNAs with advanced silencing efficacy (99). Incorporation of a modest number of LNA modifications significantly prolongs the half-life of serum siRNAs (100). SiRNAs can also be packaged in synthetic carriers to enter the cell membrane (101).

### Lentiviral Vector

Viral vectors were one of the earliest engineered nanoscale delivery systems used for transporting RNA products to targeted tissues. Viral vectors are known for their efficient transfer of codes to the cell interior and capability for escaping immunosurveillance by infected cells (102). In tumor treatment, lentiviral vectors have an advantage over other viral vectors. As a retroviral vector, the lentiviral vector avoids the drawback of transient transgene expression in the case of AdVs (102); reagent RNAs can be reversely transcribed into DNA with the help of reverse transcriptase and inserted into the genes of the targeted cells, which enables the RNA reagent to exert continual therapeutic effects on the fast-growing cell lines. It is less likely to cause an intense inflammatory response and can be processed in a relatively simple way (103). Moreover, LVs, with a maximum capacity of 8 kb nucleic strands, boast a relatively high capacity compared to other viral vectors. Although problems such as possible insertional mutagenesis (104), comparatively high immunogenicity and difficult large-scale production persist (102), the utilization of siRNA packaged in modified LVs for the modulation of onco-lncRNAs and their molecular targets will be a promising treatment for cancers.

### Liposome Carrier

Compared to viral vectors, non-viral vectors are of lower transfection efficiency (105). However, the low immunogenicity and advanced safety, relative convenient and inexpensive constructing process for large scale production make the latter one attractive in clinical therapy (102). Notably, owing to the hydrophobic nature of anchored lipids, lipid based vectors prevail over other non-lipid vectors for the extremely high transfection efficiency (106). Moreover, liposomes products comprise 70% of submissions to the FDA, which also proves the safety and effectiveness of liposomes (107). Generally, the liposome is composed of neutral or cationic amphiphilic lipid units. Neutral or positively charged polar group(s) makes up the hydrophilic “head” and a hydrophobic “tail” comprise fatty acid(s) (102). Compared to neutral ones, the cationic liposomes can integrate with negatively charged RNA molecules to form electrostatic lipoplexes, which can wrap and protect RNA from degradation by serum nucleases and promote endocytosis for cellular uptake (108). Better pharmacological properties can be achieved through liposome modulation. Conjugating a poly-(ethylene glycol) (PEG)-lipid within the lipid bilayer can extend circulating period and reduce the uptake by phagocytes (109, 110).Specific ligands and antibodies can be added to the lipid for enhanced delivery specificity (111). For example, the conjugation of transferrin to nanoparticles is demonstrated to be an efficient cancer targeted strategy due to extremely higher expression level of transferrin receptor on tumor cell surface (112).

### Exosome

Notably, exosomes can also play a part in cancer treatment by working as a novel and practical biological vector. Exosomes are vesicles released by cells into serums containing proteins, lipids and nucleic acids (113). They are involved in extracellular communication and mediate tumor progression via the contents within. As a biological vector, exosomes are less likely to activate the immune response and are highly stable *in vivo* (114), thereby prolonging their circulation duration. Their small sizes (30–150 nm) also enable them to function inside dense tissues such as osteoblasts. The general construction progress comprises five steps (114): (a) find a suitable parental cell for exosome vector production. For example, immature dendritic cells can produce exosomes that are deficient in T-cell activating factors so that they can cause minimal immune reaction (115). HEK293T cells are credited for high transferring efficacy as they can produce exosomes in large quantities. The produced exosomes can easily diffuse with targeted cells and release the inner therapeutic contents (116); (b) transfect the parental cells with plasmid containing the gene code of ligand proteins which can bind the receptor on targeted cells. In this way, exosomes are engineered with the desired ligands on their surface and they can specifically target the recipient cells. In previous practice, HEK293T cells were transfected with pDisplay encoding GE11, a ligand matching the receptors on recipient breast cancer cells, for improved targeting efficacy (117); (c) isolate the exosomes by ultracentrifugation or use of commercial kit, etc.; (d) package the therapeutic reagents into exosome vectors via electroporation; (e) inject the exosome vector into human internal environment, and the exosome can circulate *in vivo* and find its way to the target cells.

Alvarez-Erviti et al. pioneered the practice of applying engineered exosomes to deliver siRNA. They build neuronal cell-targeted exosomes and use them to pass through the blood-brain barrier and treat Alzheimer's disease (118). A recent trial using exosome vector delivering siRNA was conducted in HER2 positive breast cancer cells and BC cells (119). Although methods of exosome separation and exosomal carrier construction need considerable improvement, all these successful practices remark a bright prospect for therapeutic exosome vector.

To date, researches on targeting DANCR for cancer therapy remains limited. A previous study presented that the relative enrichment of the enzymes responsible for RNA degradation vary between cellular compartments, so the location of lncRNA can impact the suppressing efficacy of the molecular drugs on it. Comparatively, ASO is more capable of clearing the nuclear lncRNAs while RNAi have a better suppressive effect on lncRNAs in cytoplasm (120). Referring to this, the RNAi therapy is more suitable for the cytoplasmic oncogenic lncRNA DANCR (120). Moreover, being effectively suppressed by all 28 RNAi regents tested in the experiment further demonstrated that DANCR can be an ideal therapeutic target (120). Researchers should work on the construction of superior vector of the RNAi regents for better targeting effect. Remarkable progress has been made by Vaidya et al. who successfully constructed a non-viral nanoparticle carrier containing siDANCR and proved its repressive effect on the invasion and proliferation of TNBC cells via null mice injection (12). Overall, DANCR targeted therapy is of great promise and must be investigated further.

## Conclusions and Further Directions

The review has shown the vital research value of DANCR. DANCR is also a critical oncogenic regulator which presents an increasingly important status in cancer study. It can regulate hallmarks of various cancers, indicate their progression and clinical outcomes and serve as a novel target for cancer targeted treatment. Researches on DANCR remain limited and there is an urgent need for further study on this critical onco-lncRNA.

The recent progress on RNA interaction identification method includes the refined variants of immunoprecipitation techniques (such as PAR-CLIP, HITS-CLIP Maps, iCLIP, hiCLIP, CLASH etc.) and new high-throughput RNA interactome analysis methods [such as Psoralen analysis of RNA interactions and structures (PARIS), sequencing of psoralen-crosslinked, ligated, and selected hybrids (SPLASH), ligation of interacting RNA followed by high-throughput sequencing (LIGR-seq), and MARIO] (121). Without any form of crosslinking, proximity proteomics is a novel method for RNA-protein interactions studies (122). Wide application of these techniques and further development of the new ones in the late future may bring forward a new impetus for the understanding of the diverse and complicated regulatory mechanisms of lncRNA in cancers. Also, advanced techniques are in demand for the lncRNA targeted therapy. Improved targeting methods and drug vectors are needed to reduce untoward effect and improve the efficacy and specificity of the therapy.

## Author Contributions

S-JJ conceived, wrote the manuscript, and completed the figures and tables. M-ZJ revised the manuscript. W-LJ and B-RX conceived, organized, and edited the text.

### Conflict of Interest

The authors declare that the research was conducted in the absence of any commercial or financial relationships that could be construed as a potential conflict of interest.
